# Challenges to the implementation of International Health Regulations (2005) on Preventing Infectious Diseases: experience from Julius Nyerere International Airport, Tanzania

**DOI:** 10.3402/gha.v6i0.20942

**Published:** 2013-08-16

**Authors:** Edith Bakari, Gasto Frumence

**Affiliations:** 1 Ministry of Health and Social Welfare, Dar es Salaam, Tanzania; 2 Department of Development Studies, School of Public Health and Social Sciences, Muhimbili University of Health and Allied Sciences, Tanzania

**Keywords:** International Health Regulations (IHR) (2005), implementation, challenges, Port of Entry (POE), Tanzania

## Abstract

**Background:**

The International Health Regulations (IHR) (2005) is a legal instrument binding all World Health Organization (WHO) member States. It aims to prevent and control public health emergencies of international concern. Country points of entry (POEs) have been identified as potential areas for effective interventions to prevent the transmission of infectious diseases across borders. The agreement postulates that member states will strengthen core capacities detailed in the IHR (2005), including those specified for the POE. This study intended to assess the challenges faced in implementing the IHR (2005) requirements at Julius Nyerere International Airport (JNIA), Dar es Salaam.

**Design:**

A cross-sectional, descriptive study, employing qualitative methods, was conducted at the Ministry of Health and Social Welfare (MoHSW), WHO, and JNIA. In-depth interviews, focus group discussions (FGDs) and documentary reviews were used to obtain relevant information. Respondents were purposively enrolled into the study. Thematic analysis was used to generate study findings.

**Results:**

Several challenges that hamper implementation of the IHR (2005) were identified: (1) none of the 42 Tanzanian POEs have been specifically designated to implement IHR (2005). (2) Implementation of the IHR (2005) at the POE was complicated as it falls under various uncoordinated government departments. Although there were clear communication channels at JNIA that enhanced reliable risk communication, the airport lacked isolated rooms specific for emergence preparedness and response to public health events.

**Conclusions:**

JNIA is yet to develop adequate core capacities required for implementation of the IHR (2005). There is a need for policy managers to designate JNIA to implement IHR (2005) and ensure that public health policies, legislations, guidelines, and practice at POE are harmonized to improve international travel and trade. Policy makers and implementers should also ensure that implementation of the IHR (2005) follow the policy implementation framework, particularly the contextual interaction theory which calls for the availability of adequate resources (inputs) and well-organized process for the successful implementation of the policy.

In the 1800s, the global community recognized the potential spread of diseases (particularly cholera, plague and yellow fever) across international borders. Quarantine was used to prevent the spread of these diseases across international borders. This was built on the first International Sanitary Convention of 1892, which later became the International Sanitary Regulations. WHO Member States adopted this convention in 1951 (1). The regulations were revised and renamed the International Health Regulations (IHR) in 1969. The IHR (1969) were used by the WHO to direct member states on international prevention and control of infectious diseases. Under IHR (1969), the WHO Member States were also supposed to inform the WHO of plague, yellow fever, and cholera outbreaks in their areas of jurisdictions. The IHR (1969) required international passengers traveling from infected and non-infected areas to show a certificate of vaccination to the airport health authorities. During this period, ships and aircrafts were also disinfected to ensure passengers were protected against infectious disease (2).

Due to the increased risk of international spread of public health risks and hazards, the IHR (1969) were found to be insufficient in dealing with the rapid emergence of new diseases. Thus, the regulations underwent several amendments and transformations in 1978 and 1981 and the latest revision was carried out in 2005. The contents of the IHR (2005) focused at broadening its application to reflect the global disease surveillance, alert, and response (1) and harmonizing the protection of public health and ensuring the smooth operation of international travel and trade (2). These regulations require all WHO Member States to develop effective global alert, surveillance, and response strategies on prevention, protection, and control against the international spread of diseases as well as avoiding public health risks, which leads to unnecessary interference to the international traffic and trade (2, 3).

The IHR (2005) further states that the member states should put in place core capacities to facilitate smooth implementation. The core capacities include the national legislation, policy and financing, coordination and national focal point communication, surveillance, response, preparedness, risk communication, human resources, and laboratory. The designated points of entry (POEs) should have the stated core capacities all the time for controlling and responding to events of the public health emergency of international concern (2, 4).

The POEs are challenging places to work as they involve the variety of transportation of items and people from different areas of the world with their different cultures. This movement demands 24-h health-related services in all types of the weather. Therefore, appropriate health and safety measures to manage the associated risks need to be instituted at any POE (4). Airport authorities and other POEs are supposed to collaborate closely with health authorities in public health surveillance in order to identify health-related issues and trace the causes in order to control and prevent them. Identifying sources of infections among air travelers can be challenging as it can be scattered quickly upon arrival. Therefore, it is necessary to strengthen the surveillance system at the POE. Health services at POE do monitor and evaluate a range of items as well as people entering or leaving the country. It controls and prevents infectious diseases transmitted through passengers, vessels, cargo, as well as water and foodstuffs (5).

Furthermore, the contents of the IHR (2005) particularly Section IV states key tasks that each competent authority at designated POEs should perform in order to control and prevent infectious diseases. These tasks include: determining control measures to prevent local and international spread; conducting laboratory analysis and logistical assistance, for example, equipment, supplies, and transport; providing a direct link with other key players; and communicating and informing available health facilities, POE and other key operational areas for dissemination of information and recommendations from the WHO and in other countries. Other key tasks include establishing, operating, and maintaining a national public health emergency response of multi-disciplinary and multi-sectoral on public health events of international concern; and providing 24-h/7 days a week (24/7) health services at each POE (3).

Standard Operating Procedures for POEs insist on the presence of authorities with adequate capacity to respond to public health, planning for emergency of international concerns, and meet the health needs of travelers, both in-coming and out-going (6). Many countries take various measures in their POEs to prevent infectious diseases resulting from international travel and trade. In Nigeria, health officers at POEs have among others, the responsibility of taking a control measure over the infected or suspected ships arriving from a foreign port or out-going ships (7). The Indian government instructed all Port Health Officers to screen all international passengers and crews coming into India. In the case of a passenger suspected of having a flu-like illness, they should be isolated for further clinical medical examination. These measures were aiming to prevent the epidemic of influenza A H1N1 (8).

In Tanzania, a report from the Ministry of Health and Social Welfare (MoHSW) on Integrated Disease Surveillance and Response (IDSR), show that the country has experienced several outbreaks and infectious diseases from within Tanzania and from neighboring countries. Some of outbreaks and infectious diseases include measles, cholera, rift valley fever, as well as influenza A (H1N1) due to interrelation and trade along the borders and also refugees, resulting from civil and political instability in the neighboring countries (9). Tanzania, similar to other WHO Member States, is bound by all international regulations and resolutions and is expected to adopt the new initiatives. Tanzania is also a center of international travel and it is vulnerable to many international diseases and other cross-border health threats. There are 42 official POEs including airports, sea ports, lake ports, and dry ports (Table 1). These are the special areas that need to have good organization and coordination for the implementation of IHR (2005). Due to enhanced interaction through business and other developmental activities, there is the potential for diseases to spread from one area to another if prevention and control measures are not well organized (10). In Tanzania, the communicable diseases have been given a special attention in public health. The country has established a routine surveillance system: IDSR to monitor notifiable diseases, such as cholera, plague, yellow fever, rift valley fever, Ebola and avian influenza (11).

**Table 1:** List of points of entry in Tanzania

Types of points of entry	Locations of official points of entry
Sea ports (9)	Dar es Salaam, Mtwara, Tanga, Lindi, Kilwa, Mafia, Kilwa Kivinje, Bagamoyo, Pangani
Lake ports (13)	Mwanza, Nansio, Kemondo Bay, Mbambabay, Karema/Ikala, Karagonja, Kigoma, Musoma, Bukoba, Itungi, Kabwe, Kirando, Kasanga
Airports (7)	Julius Nyerere International Airport, Kilimanjaro International Airport, Songwe Mbeya, Mtwara, Mwanza, Kigoma, Tabora
Ground crossings (16)	Horohoro, Namanga, Kyaka/Mtukula, Himo/Holili, Tarakia, Sirari, Rusumo, Manyoni, Kasuanalu, Kambanga, Msimbati, Tunduma, Marongo, Kasesya, Mugaana, Marusagamba

All 194 WHO Member States agreed to implement the IHR (2005) since it came into effect on June 15, 2007. It was agreed to develop and strengthen core capacities at chosen international ports, airports, and ground crossings by June 2012. This legal instrument contains rights and obligations for countries with regard to prevention, surveillance, and response health measures applied to international travelers at POEs. Articles 5 and 13 of the IHR (2005) state that each country shall develop, strengthen, and maintain IHR (2005) at the country's earliest convenience, but not later than five years from the entry into force of this regulation, the capacity to respond well and quickly to a public health event of international concern (12). The implementation of these regulations is at different stages in the various countries, which face different challenges with the implementation of IHR (2005). Common challenges include a lack of scientific knowledge and capacity to enforce public health measures, which hinder the prevention and control of outbreaks in most developing countries (11). However, little has been documented on the challenges facing the implementation of IHR (2005) at POEs, particularly at airports in Tanzania. Thus, this study was an attempt to fill this gap by exploring and documenting the challenges facing the implementation of IHR (2005) at Julius Nyerere International Airport (JNIA) in Tanzania.

## Policy implementation framework: a contextual interaction theory

In order to realize the aim of this article, which is to explore the challenges facing the implementation of IHR (2005) in Tanzania, we utilized the contextual interaction theory, which was created by the Dutch policy researcher, Hans Bressers, in the 1990s. This theory was developed for environment protection policies indicating a need for the involvement of key actors in the implementation process. Later on, it was developed further to encompass more actors and cover broad-scale policies in various sectors, including health (13, 14).

The contextual interaction theory focuses on policy implementation and considers policy processes as the interface between actors. The policy implementation processes are controlled by actors’ activities and the type of interactions involved (15). The execution of any policy is therefore an interactive and self-motivated process, whereby actors can participate as implementers, or as target groups. Contextual interaction theory does not only consider key actors in the policy implementation process. It may also involve other stakeholders who may have an important role to play to make the implementation process a successful activity (16).

**Fig. 1:**
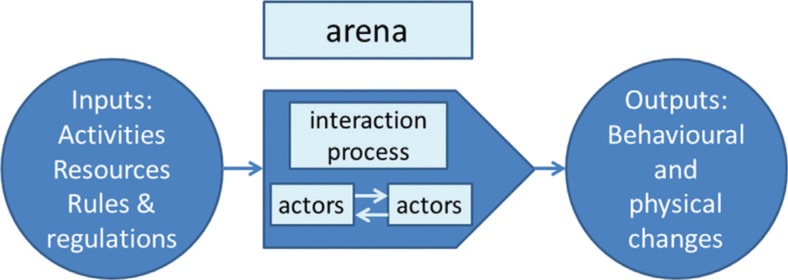
Simple model of conceptual framework of contextual interaction theory, showing conversion of inputs into outputs through an interactive process. (Modified from Bressers’ (17) work on Contextual Interaction Theory.)

The contextual interaction theory emphasizes policy implementation as a multi-actor process, involving interaction between the key parties participating in the process itself, particularly the implementers and the target groups who decide the path and outcomes of the process. The policy implementation process involves three important components (Fig. 1). The first component is the inputs, which includes activities involved and resources required for the implementation of a policy. The second component is the process, which implies a conversion process produced by the interaction of various actors and activities during the policy implementation process. The third component is the outputs, which is the outcome of the process in the form of behavioral or physical change. The output of any policy depends on the assessment of the contribution of the policy goals. The interactions are done in an environment (arena), in which rules and regulations of actions, various issues, and actors may be precisely specified or defined to facilitate policy implementation process (17).

Our study follows the current development line of contextual interaction theory in a sense that it allows the researchers to explore various inputs and actors involved in the implementation of IHR (2005) and assess other contextual factors facilitating or inhibiting the implementation process and the expected outputs of IHR (2005).

## Methods

### Study design

We conducted a descriptive, cross-sectional assessment that employed qualitative methods, particularly in-depth interviews and focus group discussions (FGDs), which allowed us to capture informant perceptions, experiences, and understanding of the challenges associated with the implementation of the IHR (2005) at POEs. This enabled us to discuss, clarify, and compare the existing situation with required standards of IHR (2005).

### Study area

#### Julius Nyerere International Airport

JNIA, the busiest POE in Tanzania, was selected due to its large inflow of international passengers (18). The airport is located on the Eastern cost of Tanzania in Dar es Salaam city, the largest commercial center in the country. It borders Zanzibar and the Indian Ocean to the East, Tanga region to its North and Coast region to its North, West, and South. According to the 2012 National Population and Housing census, Dar es Salaam has a total population of 4,364,541 people with an average annual growth rate of 5.6% (19).

#### National MoHSW

At the national level, convenience samples of four sections of the MoHSW were visited to assess the challenges associated with the implementation of IHR (2005). These sections included Environmental Health Services, Epidemiology and Disease Surveillance, Health Education and Promotion, and Emergence Preparedness and Response sections. The management of JNIA and the WHO disease surveillance unit were also purposively involved in the study in order to get their views on the challenges facing the implementation of the IHR (2005).

### Data collection

Data were collected between April and May 2012, using an in-depth interview guide. An FGD guide, focusing on the research objectives and questions, was developed and used to steer discussion with JNIA health workers. Both key informant interviews and FGD guides were developed in English and translated in Kiswahili to minimize language barriers during data collection. Documents and reports relevant for the assessment were reviewed to obtain necessary information for the study. The in-depth interviews were conducted with 11 senior national officials from MoHSW, JNIA, and WHO country office. On average, each interview lasted approximately 70 min. Two FGDs with health workers at JNIA were conducted, each lasting approximately 80 min. The first FGD consisted of five participants and the second FGD consisted of four participants, who were purposively selected to give insights into their experiences of implementing IHR (2005). FGD participants included a large proportion of health workers who were available at their workstation during the time when this study was conducted.

Additionally, researchers observed the extent to which IHR standards were met, including: use of personal protective equipment, availability of up-to-date action plans, availability of isolation rooms, offices for health workers at JNIA, vaccine storage, and so on. A desk review of documents and reports was also used to enrich the information collected from study participants.

### Data management

A daily review of data generated from in-depth interviews and FGD was conducted to ensure completeness and accuracy. Notes taken during data collection were checked against audio recorded and record review information. Audio-recorded cassettes were labeled and given unique codes to ensure easy identification by the respondent. Recorded information was then transcribed verbatim and the transcripts were filed electronically under unique codes. Missing information identified at this stage was requested from the respective respondents.

### Data analysis

In this study, thematic analysis was employed. Data were coded without necessarily fitting it into a pre-existing coding frame or the researcher's analytic preconceptions. Using an inductive approach, the themes identified are not imposed by the researcher but instead, emerge strongly linked to the the data themselves (20). The researchers analyzed data manually through reading and re-reading the transcripts until they had a general understanding of the content. As they were reviewing the transcripts, they coded the data by writing down words/phrases that captured emerging concepts. These concepts were further analyzed to identify their similarities and differences as well as identifying the main emerging themes based on the research objective.

## Results

The presentation of the results is structured by the thematic areas on the challenges facing the implementation of IHR (2005) at JNIA. Findings are based on the analysis of the responses and experiences of the respondents toward the implementation of IHR (2005) at JNIA POE. Identified themes include: low understanding of IHR (2005); poor dissemination of the national legislations and guidelines for implementation of IHR (2005); lack of clear information among officials on the designated POE for implementation of IHR (2005) in Tanzania; lack of clear coordination of plans for the implementation of IHR (2005) at JNIA; limited access to information on IHR (2005) among implementers; lack of budget allocation for emergency preparedness plans at JNIA; lack of training and orientation to health workers at JNIA; weak laboratory network to respond to the implementation of IHR (2005) at JNIA; and a shortage of financial and material resources at JNIA.

### Low understanding of the IHR

Respondents from the WHO had a clear understanding of IHR (2005). They explained that IHR (2005) are regulations put forward and agreed by WHO Member States to prevent and protect against international spread of diseases. One of the WHO respondents further elaborates ‘The IHR (2005) are the regulations that aim at controlling the transmission of infectious diseases and other events of international health concerns’ (Key Informant 9). However, some of the respondents, particularly those working with MoHSW and JNIA, had little information or understanding and were unsure about the objectives of IHR (2005) as expressed by one of the respondents from MoHSW: ‘The regulations are for the implementation of health issues at POE and may be that it has been established to control the spread of diseases from outside but I am not sure …’ (Key Informant 3).

### Poor dissemination of the revised guidelines toward the implementation of IHR (2005)

Study respondents stated that the WHO IDSR guidelines tool had been revised to encompass the requirements of IHR (2005) since 2009. However, the revised document had not yet been disseminated to users. Findings show that the health workers at JNIA were conversant with older IDSR tool, but some of them were not able to link it with IHR (2005), as mentioned by a respondent from MoHSW: ‘we know that we are required to report weekly to MoHSW using IDSR tool, but is that the IHR requirements or it is just a normal international practice of IDSR strategy?’ (FGD 2: Participant 4).

Nevertheless, one respondent insisted on the dissemination of the revised IDSR, which has incorporated the emphasis on the IHR (2005) because IDSR is an appropriate approach toward the implementation of IHR (2005) in strengthening surveillance. Respondents underscored that the main challenge for the delayed dissemination of the revised IDSR was due to lack of funds: ‘we have managed to revise the IDSR to incorporate the IHR (2005) requirements … but lack of dissemination funds is a big challenge … we are currently looking for funds to disseminate to users and implementers’ (Key Informant 1). A WHO respondent had a similar comment, noting that one of the major limitations in disseminating IHR (2005) could be attributed to the lack of adequate resources: ‘Lack of funds hinders the dissemination of the IHR (2005) making advocacy of these regulations more difficult’ (Key Informant 10).

### Lack of clear information among officials on the designated POE for implementation of IHR (2005) in Tanzania

Tanzania has multiple POEs including airports, sea ports, lake ports, and ground-crossing points as shown in Table 1. However, it was not clear how many POEs have been designated to implement IHR (2005). Many respondents mentioned different and contradictory numbers when asked how many POEs have been designated to implement IHR (2005) in Tanzania. In response, some study participants mentioned all three major POEs: Kilimanjaro International Airport, JNIA, and Mwanza Airport. One respondent claimed that there are more than 12 designated POEs, while another respondent occupying a senior position at the MoHSW said that there are no POEs in the country that met the standards of IHR (2005).

… in Tanzania, we do not have any POE which can be designated for IHR implementation, probably we need to select and focus on one POE and direct all available resources to it …. (Key Informant 6)

Despite the lack of designated POEs for implementation of IHR (2005), health workers at JNIA reported that JNIA had provisions for the application of IHR (2005) documents, for example, the International Certificate of Vaccination or prophylaxis and Aircraft General Declaration.

### Lack of clear coordination plans for the implementation of IHR (2005) at JNIA

Study findings indicate that there are three MoHSW sections (Epidemiology, Environmental Health Services, and Emergency Preparedness and Response), which have roles to play in the implementation of IHR (2005) in various ways at POE including JNIA. The Epidemiology and Disease Surveillance Section is responsible for disease surveillance and control. The Port Health Services Unit under the Environmental Health Services Section has the role of assessing risks, preparedness, and responses. The Environmental Health and Emergency Preparedness and Response section is responsible for developing and facilitating the emergency and preparedness plan for the MoHSW. It was observed that these sections have separate plans, which are uncoordinated with regard to the implementation of IHR (2005). Respondents from the MoHSW explained how these sections respond when there is an alarm or when the public health emergency of international concern has been reported from other countries or in one of the POEs in the country:

… The Epidemiology and Diseases Surveillance Section usually is informed first on any public health emergence of international concern, but the budget for preparedness and responding is placed in Environmental Health and Emergency Preparedness and Response section. But in most cases funds are not readily available, when funds are available usually cannot suffice the estimated costs to respond to the event holistically. (Key Informant 1)

Coordination was further complicated due to the involvement of the Emergency and Disaster Preparedness Unit, housed in the Prime Ministers’ Office. This unit's primary role is to coordinate all emergency and disaster-related matters at the national level. The unit comprises multi-sectoral and multi-disciplinary members, sitting in technical committees. Additionally, it has the responsibility of mobilizing resources and coordinating all responsible technical sectors. One respondent who works with the MoHSW said: ‘sometimes we have to request funds from Emergency and Disaster Preparedness Unit of the Prime Ministers’ Office for carrying out intervention to respond to reported disease of international public health concern’ (Key Informant 2).

FGD participants emphasized the need to coordinate activities performed by various sections of the MoHSW and other ministries, including the ministry of transport, in order to strengthen the performance of Tanzania airport authority:

If the two ministries will design good coordination mechanism between various sections involved in the implementation of IHR, it will be easy for each responsible organ such as JNIA to abide on the IHR requirements including provision of isolation rooms … for examining the suspects. (FGD participant 3)

### *Limite*d access to information on IHR (2005) among implementers

It was observed that the MoHSW website had limited access to information on disease surveillance and was not a reliable means of information for health alerts. This was commented on by one respondent: ‘It is very easy to get timely public health alerts reported by other countries on the websites than getting such alerts from our own country’ (Key Informant 7). Access to the internet enhances a timely and reliable means of communication worldwide. However, some of communication facilities such as computers, printers, fax machines, and internet connection at JNIA health office were not consistently available. For instance, the printers at the JNIA health office often run out of ink making it difficult to print important documents required for communication. The WHO also uses a similar means of communication as expressed by one of the respondents:

WHO communicates on surveillance of potential diseases worldwide and even other public health events of international concern. We use several means of communication like websites, emails and text message from various sources and the most reliable source is through WHO website. (Key informant 10)

### Lack of adequate budget allocation for emergency preparedness plans at JNIA

Respondents noted that a public health emergency contingency plan exists at JNIA. This was developed to enable JNIA authorities to prepare and respond rapidly to any emergencies. The following explanation was given by one respondent: ‘… Public health emergency contingency plan is the requirement of Tanzania airport authority to every airport, however, the JNIA had never followed it up to ensure that there is preparation in place in case of event …’ (Key Informant 7).

However, respondents lamented that the developed preparedness plans at JNIA were not honored in terms of allocating specific budgets required to respond to any reported emergency. A health worker at JNIA expressed this concern during theFGD, saying ‘… we have been requesting equipment or money from the Ministry of Health and Social Welfare several times; we rarely get them ….’ (FGD 1: Participant 3).

It was observed that the isolation rooms for examining suspected or ill travelers do not have the equipment required for service provision at JNIA. Given this situation, it was noted that health workers at JNIA take suspects to select hospitals in the city, particularly privately run hospitals, which have isolation rooms that are suitable for dealing with suspect cases.

It was also reported that there was only one ambulance at JNIA for health workers to facilitate the transport of suspected, ill travelers, and/or specimens to nearby health facilities. However, this ambulance was not reliable because of poor maintenance leading to difficulties in transporting suspects to the nearby health facilities.

### Lack of training and orientation to health workers at JNIA

It was noted that there was no training program for JNIA health workers for career development, knowledge, and skills updation. One respondent complained that she had not received additional training following her basic education: ‘… since I graduated I got no additional training …. I really need further training to be able to define and detect diseases appropriately …’ (FGD 1: Participant 1).

Another respondent said: ‘… It is not easy to get training opportunity by following appropriate procedures … it is mainly individual efforts to look for training opportunities …’ (FGD 1: Participant 3).

Study findings revealed that health workers and health managers, particularly those who are around the site of events, are often the ones accessing orientation programs particularly in the case definition of specific disease. It was reported that, due to limited resources, it has been difficult to orient everybody on the system. In most cases, orientations were provided to the Council Health Management Teams (CHMT), Regional Health Management Teams (RHMT), health workers at POE and tutors of health training institutions around the site of the event. In support of this argument, one respondent from MoHSW said ‘… It is very expensive to orient everybody, but when there is an alert, for instance the case of yellow fever in Uganda, the RHMT, CHMT and workers at POE around the border of Tanzania with Uganda were reoriented on the control and preventive measures as well as on the case management’ (Key Informant 4).

### Weak laboratory network to respond to the implementation of IHR (2005) at JNIA

In Tanzania, the laboratory service network was reported as a challenge for rapid response. The diagnosis and confirmation of diseases depends on the capacity of laboratories from the dispensary to national laboratories. Study respondents reported that in Tanzania, there are only two well-equipped laboratories and the Chief Government Chemist, who bear the responsibility of investigating chemical hazards. All three laboratories are located in Dar es Salaam. Other laboratories at regional and district hospitals were available to initiate investigation procedures. However, these laboratories do not have the capacity to investigate and confirm the reported health conditions. Additionally, not all specimens sent to Dar es Salaam can be analyzed there –at times, complicated cases are sent outside the country, particularly to Kenya and South Africa for further investigation. As JNIA is situated in Dar es Salaam, laboratory services may not be a problem compared to other POE. However, study respondents suggested that coordination and communication should be harmonized between JNIA and respective laboratories for timely responses of any suspects requiring laboratory investigation – ‘there is a need to have a clear network and good collaboration between health workers at JNIA and hospitals where we take specimen in order to improve the investigation process’ (FGD 2: Participant 3).

### Shortage of financial and material resources at JNIA

Most respondents reported a critical shortage of financial resources and equipment at JNIA. Reviewed reports also indicated that funds allocated to the Port Health Services Unit at MoHSW for years 2010/2011 and 2011/2012 were primarily designated for administrative and overhead expenses for 19 POE to purchase communication facilities, vehicles and motorcycles. A specific budget for JNIA was not available. For the 2011/12 budget period, less than 20% of planned funds had been disbursed at the time of this assessment. Nevertheless, during the specified period, there were no other interventions that received financial support apart from logistics and administrative services. Furthermore, it was also noted that there was a small budget allocated to different units, which are responsible for the implementation of IHR (2005); however, as reported in other sections, effective coordination between these units was lacking. Support for surveillance was obtained from the WHO and it primarily focused on technical support of national disease surveillance and in rare cases, the funds were directed to the POE.

It was observed that there were insufficient and unreliable working facilities to support the implementation of IHR (2005) including the communication facilities such as computers for accessing the internet and modern scanners to accelerate the screening of passengers entering the country through JNIA. Health workers who participated in FGDs expressed their concerns that the MoHSW has failed to provide adequate working equipment and supplies to JNIA to facilitate the implementation of IHR (2005): ‘I have been here since 1970s and lack of adequate equipment and supplies at JNIA has been a serious frustrating working situation since then …’ (FGD 1: Participant 5).

It was observed that the health office allocated to JNIA was small and unable to accommodate 20 health workers in one sitting. Additionally, the room was used as a storage facility and vaccination room for yellow fever. Study respondents reported that there was no desk allocated specifically for dealing with arriving passengers. In most cases, health workers attended to clients while standing. This situation was reported as causing occupational hazards, characterized by an unhealthy and uncomfortable working environment for individuals. The following respondent explains: ‘… we do attend arrivals while standing all the time and is like chasing and grabbing passengers … and there is no a special desk like those of customs and immigration units … we often experience back pain’ (FGD 2: Participant 4).

It was noted that the lack of adequate resources, especially funds and facilities such as isolation rooms and skilled human resources, might lead to the delay of initiatives directed toward designated POE for implementation of IHR (2005) in the country.

## Discussion

The implementation of IHR (2005) in Tanzania has faced several challenges that limit the WHO intended goal of developing systems capable of detecting and responding to any public health threat. This section is organized into the main themes that emerged from the study findings showing the major challenges facing the implementation of IHR (2005) in Tanzania: low understanding and lack of advocacy on IHR (2005); Lack of clear information among officials on the designated POE for implementation of IHR (2005); lack of clear coordination of plans for the implementation of IHR (2005) at JNIA; and lack of budget allocation for emergency preparedness plans at JNIA.

### Low understanding and lack of advocacy on IHR (2005)

Implementation of IHR (2005) requirements at POEs, and JNIA in particular, involves many stakeholders from different levels of government and institutions. However, study findings revealed that there is low understanding of the IHR (2005) among different officials, which constrain the smooth implementation of these regulations. The South East Asia regional meeting report underscored that most countries in the region have emphasized developing measures to strengthen IHR (2005) implementation at POE. However, due to a lack of awareness and training programs, their efforts have focused mainly on the prevention of infectious diseases entering into the country and have ignored other requirements of IHR (2005) (21). This study also found that there was little effort to implement advocacy programs focusing on ensuring that all ministries, departments, and agencies understand the concepts of IHR (2005). A WHO report on International Health Regulations and Aviation suggested that the dissemination of IHR (2005) requirements to all actors in the circuit enhances its implementation (4). Contextual interaction theory suggests that smooth implementation of policy requires self-motivated actors (16). The findings from this study revealed that most of the implementers of IHR (2005) are not well motivated due to lack of professional development training and advocacy programs focusing on enhancing understanding of the IHR (2005).

### Lack of clear information among officials on the designated POE for implementation of IHR (2005) in Tanzania

The IHR (2005) framework requires each WHO Member State to have at least one designated POE, which will meet the obligations of the regulations by June 2012. Several publications emphasize on the importance for the country to designate POE, develop core capacities, and identify competent authorities at each designated POE (3, 4, 22). Despite the fact that JNIA is both an international airport and the busiest airport in Tanzania, the study found that there was no designated POE in which government's efforts could focus on building the required IHR (2005) core capacities, such as surveillance, effective preparedness and response, and risk communication. Lack of designated POEs to implement IHR (2005) is contrary to the requirements of the revised regulations, which require all WHO Member States to identify the specific POEs that will comply with the IHR (2005) core capacity requirements (23). Furthermore, lack of designated POE for the implementation of IHR (2005) in Tanzania is also contrary to the policy implementation framework (the contextual interaction theory), which requires policy makers to specify an arena where interaction of actors, activities, rules and regulations will take place during the policy implementation process (17).

### Lack of clear coordination of plans for the implementation of IHR (2005) at JNIA

According to the WHO, the IHR (2005) article 4 mandates the member states to appoint IHR national focal point agencies for coordinating IHR implementation in the country (1). Good coordination networks have been proposed as the best solution to the implementation of policy, program, or project. A well-organized and coordinated network helps actors in the policy implementation and in achieving the organizations’ end results (24). Despite the importance of IHR (2005) requirements at POEs, findings from this study revealed weak coordination among key stakeholders on the focus of building required core capacities at JNIA. Within the MoHSW, the structure seemed to be disintegrated in fostering health services at POE, particularly at JNIA. Lack of appropriate and frequent coordination during the implementation of any policy may lead to friction and conflict within the organization and this situation may partly be caused by a lack of clear understanding of actors’ roles and responsibilities (24). In this study, findings have indicated that in Tanzania there are different authorities responsible for the implementation of IHR (2005). However, the roles and responsibilities of these authorities are not only unclear but there are also unstructured communication channels between implementing organs of IHR (2005). Lack of structured communication channels between IHR national focal point, the WHO, and other sectors complicate the implementation of IHR (2005) particularly in risk communication.

Contrary to our findings, in Uganda, a study on the assessment of core capacity for the IHR (2005) found that despite a lack of national guidelines on risk communication to back-up the information, the country has a well-designed channel of risk communication from the national to the district level (25).

### Lack of budget allocation for emergency preparedness plans at JNIA

The IHR (2005) strongly encourages countries to institute and strengthen the core capacities at international POEs, which includes public health emergency preparedness and response through the development of a public health emergency contingency plan (4).

Preparedness includes the mapping of potential hazards and its sites, identification of required resources and capacity to support the required operations during the public health emergency (26). The first component of the policy implementation process as described in the contextual interaction theory (17) requires actors or implementers to have adequate inputs, which include among other technology, funds, and human resources for the proper implementation of the policy. However, this study reports that the primary challenge for the implementation of IHR (2005) is the lack of adequate resources both in terms of funds, motivated and well-trained human resources as well as a lack of well-equipped isolation rooms for the implementation of IHR (2005) at POEs. A study on an assessment of mental health policy in Ghana, South Africa, Uganda, and Zambia reported that the implementation of mental health policy in poor resource settings face difficulties because some of the developing countries do not have reliable sources of financing policy implementation (27). A study that assessed global public health surveillance under new IHR (2005) reported similar findings indicating that mobilizing adequate resources to implement IHR (2005) in poor resource countries is a major challenge (28). Assessment conducted in countries in the South East Asia Region (SEAR) revealed that in order to implement the IHR (2005) at POEs, more resources are needed for training of human resources, ensuring adequate supplies and equipment, and operation services for strategic linkage with other collaborators related to IHR requirements (4). For the government of Tanzania to have a successful implementation of IHR (2005) and other policies, programs, and projects, there is a need for mobilizing and allocating adequate resources (inputs for policy implementation) such as funds, skilled and highly motivated human resources, appropriate technology, and other infrastructures including offices and transport facilities.

## Strengths and weaknesses of the study

To judge the trustworthiness of the results, the study adopted the criteria of credibility, transferability, dependability, and confirmability as used elsewhere (29). We employed triangulation in data collection by including key informant interviews, FGD, observation, and document reviews to increase credibility. The purposive sampling of key informants and FGD participants to reach maximum variation aimed at increasing transferability of the study findings. Researchers conducted mid- and end-point debriefing meetings during and after data collection to reflect and discuss procedures and interpretation of the results in order to ensure confirmability and consistency. Obtaining an adequate number of FGD participants was the main limitation of the study. It was difficult to have FGD of six or more participants because health workers at JNIA are working on different shifts so as to ensure 24-h coverage of health services. Some JNIA health workers were on annual leave and others had other important responsibilities to attend to both in and out of the office during the time this study was conducted. However, the research team ensured that FGDs that were conducted were exhaustive by exploring all issues identified in the interview guide.

## Conclusions

The study aimed at assessing challenges facing the implementation of IHR (2005) at the JNIA in Tanzania. Study findings indicate that similar to other developing countries, there are several challenges facing the implementation of various policies, programs, and projects in Tanzania. These include low understanding and poor advocacy of the IHR (2005); lack of a clear coordination system for the implementation of the regulations; limited access to information on IHR (2005) among implementers, and lack of budget allocation for emergency preparedness plans. Conclusively, it is argued that in order for policy makers and implementers of any policy to achieve the desired goals (output of the policy), they have to ensure that the other two components of policy implementation as described in the contextual interaction theory are well addressed during the policy development process. This means that inputs required for the policy implementation (adequate resources in terms of well-trained human resources, technology, finance, and equipment) must be assured, and process involved in the implementation of the policy (the arena where interaction of actors and non-actors takes place) should be well organized.
